# Reshaping Antioxidant Activity via Photoisomerization: A Comparative Theoretical Study of Pterostilbene and Resveratrol

**DOI:** 10.3390/antiox15030325

**Published:** 2026-03-05

**Authors:** Lei Wang, Chaofan Sun

**Affiliations:** College of Science, Northeast Forestry University, Harbin 150040, China; wangl@nefu.edu.cn

**Keywords:** pterostilbene, photoisomerization, antioxidant, molecular docking, DFT

## Abstract

This study elucidates the regulatory mechanisms of methoxy substitution and photoexcitation on the antioxidant properties of pterostilbene (PTE) versus resveratrol (RES), employing a combined approach of multi-reference calculations, density functional theory (DFT), time-dependent DFT (TD-DFT), and molecular docking. Spectral analysis indicates that trans isomers exhibit a significant redshift (~13 nm) and have oscillator strengths more than double those of cis isomers. A pivotal difference in photoisomerization kinetics was identified: methoxy substitution drastically lowers the isomerization barrier for RES, indicating that PTE is more readily photoisomerized. Regarding radical scavenging, thermodynamic data confirm that Hydrogen Atom Transfer (HAT) and Radical Adduct Formation (RAF) are spontaneous pathways; notably, the O1 site of trans-PTE serves as the optimal hydrogen donor. Conceptual DFT (CDFT) analysis reveals that photoexcitation triggers a dramatic electronic reconfiguration, particularly for cis-PTE, whose ionization potential in the S1 state drops sharply to 4.66 eV, accompanied by an increased softness of 0.38 eV^−1^, rendering it a highly potent electron donor. Furthermore, molecular docking demonstrates that trans-PTE robustly occupies the Keap1 Kelch pocket (binding energy: −7.478 kcal/mol) to inhibit Nrf2 binding via its favorable planar geometry.

## 1. Introduction

Oxidative stress, characterized by an imbalance between the production of reactive oxygen species (ROS) and the biological system’s ability to detoxify reactive intermediates, is implicated in the pathogenesis of various chronic diseases, including cancer, cardiovascular disorders, and neurodegeneration [1,2,3,4]. To counteract oxidative damage, organisms have two sophisticated defense systems: the direct scavenging of free radicals by small-molecule antioxidants and the indirect upregulation of cytoprotective enzymes via the Keap1-Nrf2 signaling pathway [5,6,7]. The Keap1-Nrf2 pathway serves as a master regulator of the cellular antioxidant defense. Keap1 recognizes Nrf2 through its distinct structural domains, thereby binding to it and promoting its degradation. Keap1 is a cysteine-rich protein, and within its high-affinity binding pocket, three arginine residues (ARG-380, ARG-415, and ARG483) interact with the glutamate and aspartate residues in the two Nrf2 motifs. Under oxidative stress, specific cysteine residues of Keap1 undergo oxidation, leading to conformational changes in Keap1. This subsequently results in the dissociation of the Nrf2-Keap1 complex, disrupting their interaction and allowing Nrf2 to translocate to the nucleus to activate cytoprotective genes. Therefore, designing small molecules that competitively occupy the Keap1 Kelch pocket and inhibit Nrf2 binding represents a highly promising strategy for indirectly enhancing cellular antioxidant capacity [8,9].

Among natural polyphenols, resveratrol (RES) has garnered significant attention for its potent antioxidant properties [10,11,12]. However, its therapeutic application is often limited by rapid metabolism and poor bioavailability [13,14,15]. Pterostilbene (PTE), a naturally occurring dimethoxylated analog of RES, has emerged as a superior candidate, exhibiting enhanced lipophilicity, metabolic stability, and biological efficacy [16,17,18]. Furthermore, the spatial configuration of isomers also exerts a certain influence on their biological activity. For instance, studies on conjugated systems like carotenoids have demonstrated that Z-isomers can exhibit significantly enhanced targeted biological activities—such as UV-shielding and anti-aging effects—compared to their naturally dominant all-E-counterparts, even when their baseline radical scavenging capacities differ [19]. Tchekalarova et al. (2024) demonstrated that, among cinnamic acid and caffeic acid derivatives, their cis isomers exhibited significantly stronger anticonvulsant and analgesic activities than their trans isomers [20]. A recent study on astaxanthin by Chen et al. revealed that the 9-cis isomer exhibits 1.27 times and 1.49 times greater ability to quench singlet oxygen compared to the 13-cis and all-trans isomers, respectively [21]. These findings further confirm that molecular geometry serves as the key structural basis regulating its biological effects. While the structure–activity relationships (SAR) of stilbenes in their S0 state have been extensively studied, a critical aspect of their chemical nature remains underexplored: their photo-sensitivity. Stilbene derivatives possess a central C=C double bond, making them prone to trans-to-cis photoisomerization upon exposure to UV or visible light [22,23]. This photochemical property is particularly relevant given that antioxidants often function in light-exposed environments (e.g., skin, food storage, or photodynamic therapies). An intriguing yet unresolved question arises: does photoexcitation and the subsequent geometric isomerization enhance or diminish the antioxidant capacity of these molecules? Furthermore, how does the methoxy substitution in PTE modulate the potential energy surface (PES) of this photo-process compared to the hydroxylated RES? Most conventional theoretical studies relying on single-reference Density Functional Theory (DFT) often fail to accurately describe the topology of conical intersections (CIs) and excited-state dynamics, leaving the structure–photo-activity relationship largely ambiguous.

**Figure 1:**
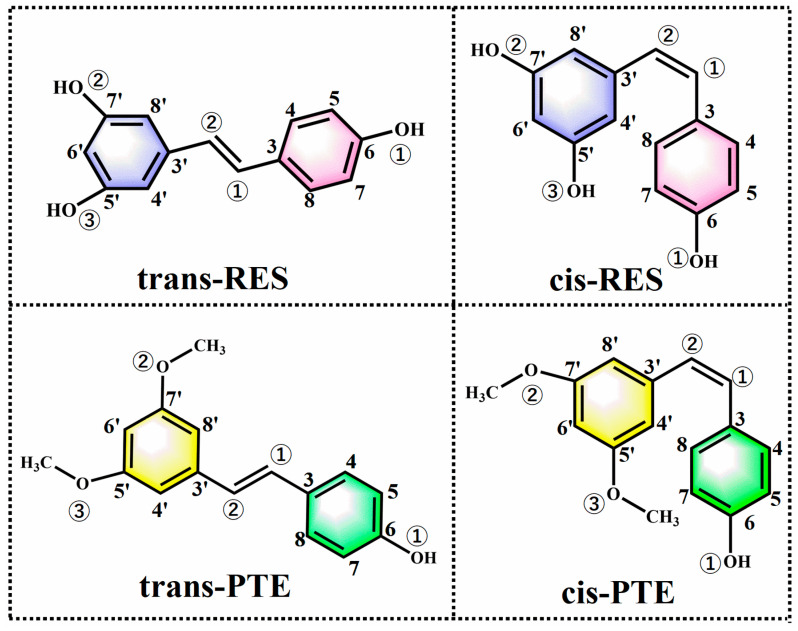
Molecular structures of the cis and trans isomers of RES and PTE.

In this context, understanding the distinct roles of substituent effects and photoisomerization is essential for elucidating the full antioxidant mechanism of PTE. In the present study, we employ a comprehensive theoretical framework combining multi-reference electronic structure calculations (CASPT2//RASSCF) with Conceptual DFT (CDFT) and molecular docking to systematically investigate the antioxidant mechanisms of PTE versus RES. Specifically, we aim to: (1) accurately map the photoisomerization pathways and quantify the energy barriers using high-level multi-reference methods; (2) elucidate how photoexcitation reshapes the electronic reactivity descriptors (e.g., ionization potential and softness) of different isomers; and (3) evaluate the potential of these isomers to inhibit the Keap1-Nrf2 interaction through molecular docking. By integrating quantum chemical insights with biological simulation, this work provides a novel theoretical basis for understanding the synergistic dual-mechanism of photosensitive antioxidants. The chemical structures of RES and PTE are illustrated in Figure 1.

## 2. Materials and Methods

### 2.1. DFT and TD-DFT Calculations

All S_0_-state and S_1_-state geometry optimizations and frequency calculations were performed using density functional theory [24,25] (DFT) and time-dependent DFT [26,27,28] (TD-DFT), respectively. These calculations employed the ω-B97XD functional and the 6-311G(d,p) basis set, as implemented in the Gaussian 16 software package [29]. UV-Vis absorption spectra were simulated based on the optimized S_0_-state geometries. Solvent effects were modeled using the integral equation formalism polarizable continuum model (IEFPCM) with methanol as the solvent. Frontier molecular orbital diagrams were visualized using VMD 1.9.4 [30].

### 2.2. Multi-Reference Calculations for Photoisomerization

To accurately describe the photoisomerization pathways and the topology of CIs, multi-reference calculations were performed using the OpenMolcas v24.06 program [31,32]. Given the limitations of single-reference methods and the potential quasi-degeneracy or strong mixing of multiple electronic states near the S_0_/S_1_ conical intersection, a four-state averaging (SA-4) approach was employed to provide a balanced description of the potential energy surfaces and to avoid root-flipping issues during the conical intersection geometry optimization. The S_0_/S_1_ conical intersection was optimized at the SA-4-RASSCF(12,12)/6-311G(d,p) level of theory [33,34]. The active space consisted of (12e, 12o), covering the entire π and π* system of the stilbene backbone. The corresponding orbital images are provided in Figures S1 and S2 of the Supporting Information. To construct the reaction pathway, a series of intermediate geometries connecting the Franck–Condon (FC) point and the CI point were generated using the image-dependent pair potential (IDPP) interpolation method. The IDPP algorithm constructs the path by prioritizing relative atomic distances and incorporating a weighting function, where closer atom pairs exert greater influence. This approach effectively prevents spurious bond breaking and unphysical atomic overlaps, yielding a smooth and physically reasonable path that approximates the minimum energy path (MEP) [35,36]. The IDPP interpolation was implemented using the ORCA 6.0.0 software package [37]. Subsequently, to recover dynamic electron correlation, single-point energy calculations were performed on each interpolated geometry along these paths using the multistate complete active space second-order perturbation theory (MS-CASPT2) method in combination with the def2-TZVP basis set. To mitigate potential intruder state issues, an imaginary level shift of 0.2 a.u. was applied in the MS-CASPT2 calculations, while the IPEA shift was set to the standard value of 0.0 a.u [38,39]. The solvent environment (methanol) was implicitly modeled using the polarizable continuum model (PCM).

### 2.3. Reactivity Descriptors and Antioxidant Mechanisms

Global reactivity descriptors based on CDFT [40], including ionization potential (IP), electron affinity (EA), chemical hardness (η), softness (S), and electrophilicity index (ω), were computed using Multiwfn 3.8 [41,42]. The key equations are defined as follows:

(1) IP=E(N−1)−E(N)

(2) EA=E(N)−E(N+1)

(3) hardness(η)=(IP−EA)/2

(4) softness(S)=1/2η

(5) $$electronegativity\left(\chi \right)=\left(IP-EA\right)/2$$

(6) chemical potential(μ)=−electronegativity(χ)

(7) $$electrophilicity(\omega )=\mu^{2}/2$$

where E(N), E(N − 1), and E(N + 1) represent the energies of the neutral, cationic, and anionic species, respectively. To evaluate the radical scavenging potential, the thermodynamic feasibility of three mechanisms—Hydrogen Atom Transfer (HAT), Single Electron Transfer (SET), and Radical Adduct Formation (RAF)—was assessed by calculating the change in Gibbs free energy (ΔG) for the reaction with hydroperoxyl radicals (HOO^•^) at 298.15 K and 1 atm. Additionally, the ΔG value incorporates the zero-point energy correction term, thermodynamic correction terms at 298.15 K and 1 atm, and the solvation contribution implicitly modeled by the IEFPCM framework with methanol as the solvent. The calculation equation is as follows:

(1) $HAT:AH+{HOO}^{•}\to A^{•}+HOOH$
  $\Delta G_{HAT}=G\left(A^{•}\right)+G\left(HOOH\right)-G\left(AH\right)-G({HOO}^{•})$
(2) $RAF:AH+{HOO}^{•}\to [{AH-OOH]}^{•}$
  $\Delta G_{RAF}=G\left([{AH-OOH]}^{•}\right)-G\left(AH\right)-G({HOO}^{•})$
(3) $SET:AH+{HOO}^{•}\to {AH}^{•+}+{HOO}^{-}$
  ${\Delta G}_{SET}={G(AH}^{•+})+{G(HOO}^{-})-G(AH)-{G(HOO}^{•})$

It is worth noting that for all open-shell radical species and adducts calculated at the unrestricted Uω-B97XD level, the minor spin contamination prior to annihilation was effectively corrected through spin annihilation procedures, restoring the <S^2^> values to the ideal theoretical value of ~0.750 for a pure doublet state. Consequently, the calculated thermodynamic energies remain highly reliable.

### 2.4. Molecular Docking

Molecular docking studies were performed to investigate the binding modes of PTE and RES with the Keap1 protein. The crystal structure of the Keap1-Nrf2 complex was retrieved from the Protein Data Bank (PDB ID: 2FLU) [43]. Before docking, the Nrf2 peptide was removed to simulate a competitive inhibition scenario. The protein structure was prepared using AutoDock Tools 1.5.7 by removing water molecules, adding polar hydrogens, and assigning Gasteiger charges. The 3D structures of the ligands (cis/trans-PTE and RES) were derived from the DFT-optimized geometries. Docking calculations were executed using AutoDock Vina. The grid box was centered on the Kelch domain binding pocket (covering residues ARG-380, ARG-415, ARG483 and SER-555) with dimensions of 40 × 40 × 80 Å and a default grid spacing of 1.0 Å [44,45]. The structure exhibiting the lowest binding energy was selected, representing the thermodynamically most favorable configuration. This structure served as the basis for analyzing the inhibitory potential of the target molecules against the Keap1-Nrf2 complex. Visualization and interaction analysis were conducted using PyMOL 2.5.0 [46] and AutoDock Vina 1.2.0 [47,48].

## 3. Results

### 3.1. Absorption Spectra and Electronic Structure

**Figure 2:**
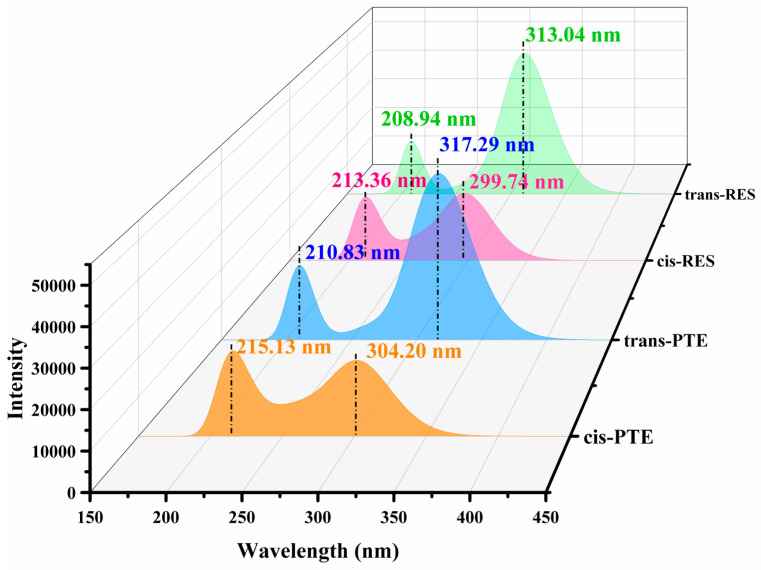
Absorption spectra illustrating the differences between the cis and trans isomers of the two molecules.

TDDFT calculations were performed to investigate the absorption properties of cis- and trans-RES and PTE. As summarized in Figure 2 and Table S1, all four molecules exhibit a dominant absorption band in the near-UV region, which is mainly attributed to the S_0_→S_1_ transition with a pronounced π–π* character [49]. In all cases, the lowest excited state is dominated by a HOMO→LUMO transition, with a contribution of approximately 68–69%, indicating that the nature of the electronic excitation remains essentially unchanged upon isomerization or substitution. A clear bathochromic shift is observed when comparing the trans isomers with their cis counterparts. For RES, the main absorption maximum shifts from 299.7 nm (cis) to 313.0 nm (trans), while for PTE, a similar red shift from 304.2 nm to 317.3 nm is obtained. This red shift is accompanied by a significant increase in oscillator strength, with the f value of the trans isomers (~1.15) being more than twice that of the corresponding cis forms (~0.45–0.48), suggesting a more allowed electronic transition in the trans configuration. Such behavior can be attributed to the enhanced molecular planarity and extended π-conjugation in the trans structures, which improve orbital overlap.

**Figure 3:**
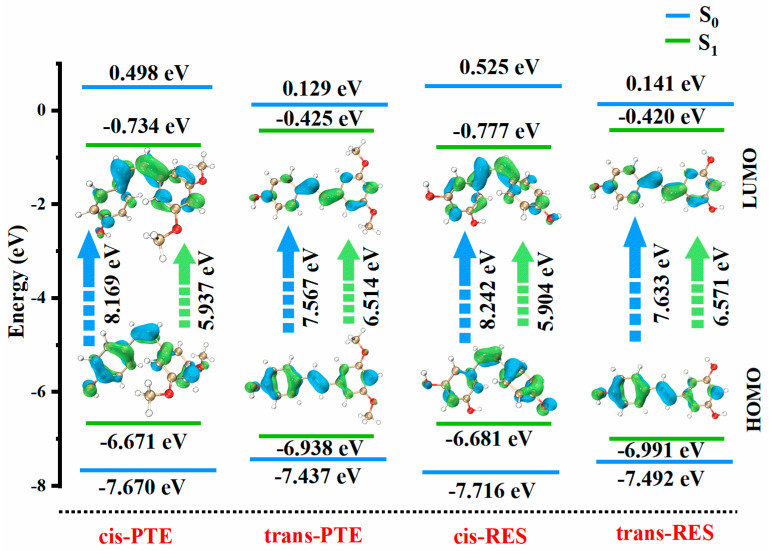
Frontier molecular orbital (HOMO/LUMO levels and ΔE) for the cis and trans isomers of PTE and RES.

In addition to configurational effects, the introduction of two methoxy substituents in PTE leads to a further, albeit modest, red shift of approximately 4–5 nm relative to RES in both cis and trans forms. This observation reflects the relatively weaker but systematic electronic influence of the substituents, which act mainly as electron-donating groups and slightly perturb the conjugated framework. The frontier molecular orbital analysis provides strong support for the above spectral trends. As shown in Figure 3, the trans isomers exhibit higher HOMO energies and lower LUMO energies than the corresponding cis forms, resulting in smaller energy gaps (RES: 7.63 eV vs. 8.24 eV; PTE: 7.57 eV vs. 8.17 eV). Meanwhile, PTE displays slightly narrower gaps than RES within the same configuration, consistent with the observed substituent-induced red shift.

### 3.2. Photoisomerization Process

**Figure 4:**
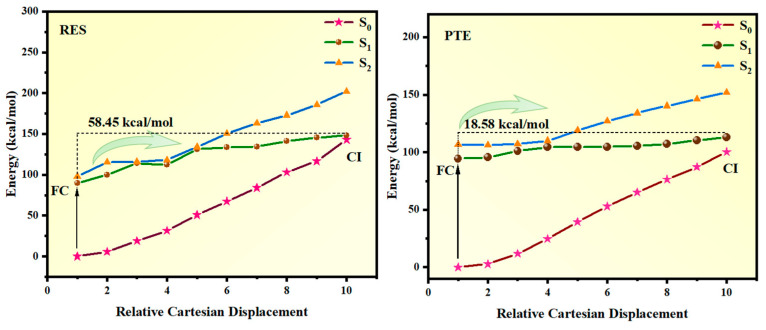
Photoisomerization from trans to CI (PTE and RES).

To elucidate the effect of methoxy substitution on the photophysical behavior of resveratrol, the photoisomerization pathways of RES and its derivative PTE were systematically investigated. For both molecules, the photoisomerization process initiates from the trans configuration. Upon photoexcitation to the first excited singlet state, the molecule evolves along the excited-state PEC and undergoes non-radiative decay back to the S_0_ state through a CI, thereby completing the trans-cis isomerization. This process is detailed in Figure 4.

An accurate description of the S_0_/S_1_ CI is crucial, as it constitutes the primary pathway governing ultrafast internal conversion during photoisomerization [50,51,52]. Owing to the pronounced multi-reference character inherent to non-adiabatic regions, a multi-reference electronic structure approach was employed. Specifically, the S_0_/S_1_ CI geometries were optimized at the SA4-RASSCF level using the 6-311G(d,p) basis set with an active space of (12e, 12o). To construct the minimum energy pathway from the trans minimum to the CI, an initial reaction coordinate was generated using the IDPP interpolation scheme. Single-point energies along this pathway were subsequently refined using the MS-CASPT2 method to obtain reliable excited-state potential energy profiles. The resulting PECs are presented in Figure 4. A pronounced difference is observed between RES and PTE upon methoxy substitution. For RES, the energy barrier for photoisomerization—defined as the energy difference between the trans minimum and the CI—amounts to 58.45 kcal/mol. In contrast, the corresponding barrier in PTE is drastically reduced to 18.58 kcal/mol. This substantial lowering of the activation barrier indicates that methoxy substitution significantly reshapes the excited-state potential energy landscape, rendering the photoisomerization process in PTE energetically more favorable and kinetically more accessible. The reduced barrier in PTE can be attributed to the electron-donating nature of the methoxy groups, which stabilizes the excited-state electronic configuration and facilitates charge redistribution along the π-conjugated framework during the twisting motion around the central C=C bond. Consequently, the system can access the CI more efficiently, leading to an enhanced non-radiative decay channel and a faster photoisomerization process compared to RES. It should be noted that these barriers represent the unrelaxed upper-bound energy penalties along the IDPP path originating directly from the FC point, rather than the true relaxed kinetic activation energies.

### 3.3. Radical Scavenging Mechanisms

To comprehensively evaluate the antioxidant activity of resveratrol derivatives, three major radical scavenging mechanisms—hydrogen atom transfer (HAT), single-electron transfer (SET), and radical adduct formation (RAF)—were systematically investigated by calculating the corresponding key thermodynamic parameters [53,54,55].

#### 3.3.1. Hydrogen Atom Transfer (HAT) Pathway

The HAT mechanism involves the transfer of a phenolic hydrogen atom to the hydroperoxyl radical (HOO^•^). The thermodynamic feasibility of this process was evaluated using the Gibbs free energy change in the HAT reaction (ΔG_HAT_), with the most reactive hydroxyl sites summarized in Table 1. Negative (ΔG_HAT_) values indicate a thermodynamically spontaneous hydrogen abstraction process. Among all examined sites, the O1 position in the four molecules exhibits the most favorable HAT thermodynamics, with ΔG_HAT_ values of −2.422 kcal/mol for trans-PTE and −2.241 kcal/mol for trans-RES. These results identify the O1 phenolic group as the preferred hydrogen-donating site and confirm HAT as a viable radical scavenging pathway.

#### 3.3.2. Radical Adduct Formation (RAF) Pathway

To determine radical addition sites, condensed Fukui functions were calculated for each atom in the four molecules, where f^0^ describes susceptibility to radical attack. The results are presented in Supplementary Materials Table S2. The highest f^0^ values for RES and PTE are predominantly located on key carbon atoms within the conjugated skeleton, particularly at sites associated with C=C fragments, indicating a stronger tendency for radical addition at these positions. Therefore, the Gibbs free energy change (ΔG_RAF_) for the addition of HOO^•^ to the C=C double bonds was subsequently calculated to evaluate the RAF mechanism. All calculated ΔG_RAF_ values are significantly negative, ranging from −7.15 to −13.01 kcal/mol, indicating that the formation of radical adducts is a highly exergonic and thermodynamically favorable process. Notably, the largest thermodynamic driving force (ΔG_RAF_ = −13.01 kcal/mol) is observed for the cis-RES system, highlighting the strong stabilization of the radical adduct in the cis configuration. This pronounced exergonicity suggests that RAF represents the thermodynamically most favorable radical scavenging pathway among the three mechanisms considered.

#### 3.3.3. Single-Electron Transfer (SET) Pathway

The feasibility of the SET-based antioxidant mechanism was evaluated by calculating the Gibbs free energy change (ΔG_SET_) for the electron transfer reaction between the antioxidant and the HOO^•^ radical. This thermodynamic parameter reflects the intrinsic electron-donating ability of the molecule and has been widely employed to compare the viability of SET mechanisms. The calculated values follow the order: cis-RES (47.27 kcal/mol) > cis-PTE (46.29 kcal/mol) > trans-RES (45.61 kcal/mol) > trans-PTE (44.72 kcal/mol). The consistently lower SET energies observed for the trans isomers indicate that the trans geometry facilitates electron donation, which can be attributed to enhanced π-conjugation and the higher HOMO energy level in the trans-configuration, both of which facilitate electron transfer. Among all the studied systems, trans-PTE exhibited the lowest ΔG_SET_ value, indicating its strongest thermodynamic propensity to achieve radical scavenging via the SET mechanism.

It should be noted that this analysis primarily focuses on the thermodynamic feasibility of the radical scavenging pathways. Although the RAF mechanism exhibits the highest exothermicity, the actual reaction rates are governed by activation energy barriers. Due to structural complexity and the diversity of potential reaction sites, the kinetic competition between the HAT and RAF pathways remains to be further elucidated. Therefore, the term ‘favorable’ in this study strictly refers to the thermodynamic driving force.

### 3.4. Global Descriptive Descriptors

**Figure 5:**
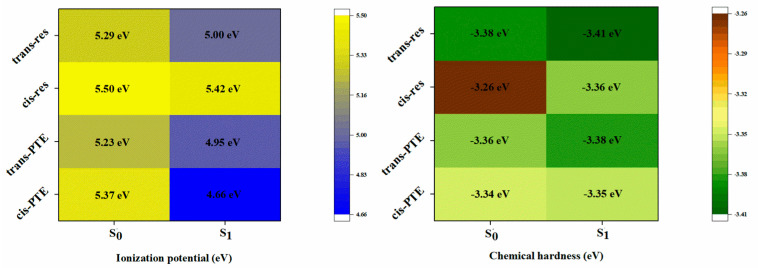
Ionization potentials and chemical hardness of several molecules.

To elucidate the differences in antioxidant behavior between PTE and RES, conceptual density functional theory (CDFT) was employed to calculate a set of global reactivity descriptors for both molecules in the S_0_ state and the S_1_ state, including the ionization potential (IP), electron affinity (EA), chemical hardness (η), softness (S), and electrophilicity index (ω) (Figure 5 and Table S3).

The IP is a key descriptor for evaluating the ability of a molecule to scavenge free radicals via the SET mechanism, with lower IP values indicating stronger electron-donating capability [56,57]. The calculated results show that trans-PTE exhibits a lower S_0_-state IP value (5.23 eV) than trans-RES (5.29 eV), and a similar trend is observed for the corresponding cis isomers. This reduction in IP can be attributed to the strong electron-donating inductive effect of the methoxy substituents in PTE. The introduction of methoxy groups increases the electron density of the conjugated framework, facilitating electron removal from the HOMO. From an electronic-structure perspective, these results indicate that PTE possesses a superior intrinsic electron-donor character compared to RES, which is consistent with the lower ΔG_SET_ values obtained in the SET mechanism discussed above. A comparison of configurational effects further reveals that, in the S_0_ state, the trans isomers of both molecules exhibit lower chemical hardness and higher softness than their cis counterparts. For example, the softness of trans-PTE (0.27 eV^−1^) is notably higher than that of cis-PTE (0.25 eV^−1^). This suggests that, in the S_0_ state, the trans configuration represents a more efficient antioxidant. In contrast, upon light-induced trans→cis isomerization, the increase in IP and η implies a potential weakening of antioxidant activity [58,59].

The S_1_-state results demonstrate that photoexcitation markedly amplifies the electronic-structure differences arising from both configurational and substituent effects. In general, all systems exhibit reduced IP values and increased softness in the S_1_ state, indicating a dramatic photo-induced electronic activation upon photoexcitation. However, the magnitude of this enhancement varies significantly among the different molecules. With respect to configurational effects, the cis isomers display a more pronounced electronic activation in the S_1_ state than the corresponding trans isomers, particularly in the case of PTE. Notably, the IP value of cis-PTE decreases sharply from 5.37 eV in the S_0_ state to 4.66 eV in the S_1_ state, accompanied by a substantial reduction in chemical hardness to 2.62 eV and an increase in softness to 0.38 eV^−1^. These changes indicate an exceptionally high electronic polarizability and a strong theoretical photoinduced electron transfer in the S_1_ state. By contrast, although trans-PTE also exhibits electronic activation upon photoexcitation, the extent of these changes is significantly smaller than that observed for the cis isomer. This finding suggests that light-induced trans→cis isomerization does not merely alter the molecular geometry but fundamentally reshapes the electronic reactivity of the system.

In terms of substituent effects, a clear distinction between PTE and RES is observed in the S_1_ state. While moderate differences already exist in the S_0_ state, photoexcitation dramatically amplifies the influence of methoxy substitution in PTE, resulting in significantly lower IP values and chemical hardness, as well as higher softness, compared to the corresponding RES systems. In particular, cis-PTE exhibits the lowest IP and the highest softness among all species studied, highlighting its potent character as a transient super-electron-donor. Overall, the CDFT descriptors demonstrate that photoexcitation, through the synergistic interplay of configurational transformation and substituent effects, substantially enhances the electronic reactivity of PTE, especially in its cis configuration. Given the ultrafast nature of internal conversion and photoisomerization (typically on the femtosecond to picosecond timescale), direct diffusion-controlled bimolecular radical scavenging from the S_1_ state is kinetically unfavored. Therefore, the sharply reduced IP and enhanced softness in the S_1_ state should be interpreted as theoretical indicators of intrinsic photo-electronic activation rather than describing a sustained reactive state.

### 3.5. Molecular Docking Analysis

Molecular docking is a well-established computational method in drug discovery, widely employed to predict ligand–target interactions and binding affinities, thereby facilitating the identification of therapeutic candidates [60,61,62,63]. In biological systems, a complex antioxidant defense network maintains redox homeostasis. Notably, many antioxidant processes and enzymes are directly regulated by the Keap1-Nrf2 pathway [63,64,65]. Consequently, the exploration of small molecules capable of inhibiting the formation of the Keap1-Nrf2 complex is of significant importance in antioxidant research.

**Figure 6:**
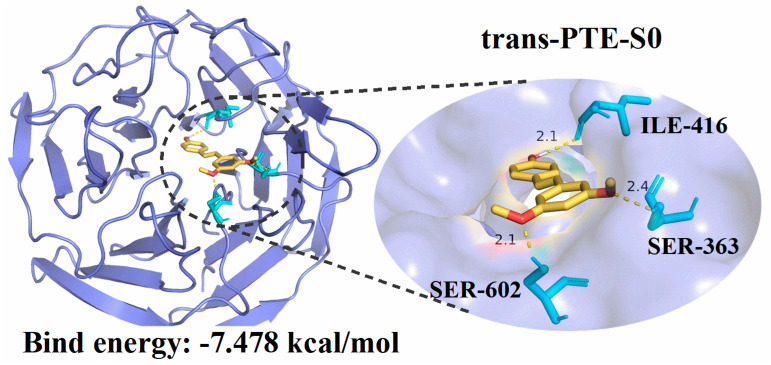
Molecular docking simulation illustrating the predicted binding mode of trans-PTE within the active site of the Keap1 protein.

In this study, molecular docking was performed to assess the potential of PTE and RES to occupy the Keap1 Kelch pocket [66]. As illustrated in Figure 6 and Supplementary Figures S3–S5, both PTE and RES can bind deeply within the Kelch domain pocket of Keap1. The calculated binding affinities for all systems fall within a reasonable thermodynamic range of −6.0 to −7.5 kcal/mol, suggesting a structural basis for these compounds to act as potential Keap1 inhibitors. Crucially, the ligands form stable hydrogen bonds with key residues such as ARG-380, ARG-415, and SER-555, which are identical to the residues responsible for anchoring the Nrf2 peptide [45,60]. For trans-PTE, the S_0_ state exhibits the most favorable binding energy (−7.478 kcal/mol), surpassing that of cis-PTE in the S_0_ state (−6.739 kcal/mol). This suggests that the extended planar geometry of the trans-configuration offers superior spatial compatibility with the deep Kelch pocket compared to the cis-form. In the case of RES, the difference between isomers is relatively small: in the S_0_ state, cis-RES (−6.283 kcal/mol) shows slightly better binding than trans-RES (−5.959 kcal/mol). Overall, the docking results confirm the potential of PTE and RES to occupy the Keap1 Kelch pocket. Specifically, trans-PTE demonstrated the most robust binding capacity. The overlap of these binding sites with the critical Nrf2 recognition residues provides a structural basis for their indirect antioxidant effects via the modulation of the Keap1-Nrf2 signaling pathway.

## 4. Conclusions

In this study, a combined theoretical approach using DFT/TD-DFT calculations and molecular docking was employed to elucidate the antioxidant mechanisms of PTE versus RES, with particular emphasis on revealing the synergistic regulatory role of methoxy substitution and photoisomerization in antioxidant activity.

First, investigations using multi-reference methods revealed that the introduction of methoxy groups in PTE effectively lowers the energy barrier for photoisomerization, facilitating the trans→cis transformation compared to RES. Analysis of global reactivity descriptors indicated that while PTE exhibits slightly superior antioxidant properties to RES in the S_0_ state, where the trans-isomer generally possesses a lower IP and thus stronger electron-donating capacity, photoexcitation dramatically reshapes this landscape. All systems display a significant reduction in IP upon photoexcitation, enhancing their radical scavenging potential. Uniquely for PTE, the cis-isomer exhibits a profound photo-electronic activation in the S_1_ state, possessing the lowest IP among all studied species. Although the ultrashort lifetime of the S_1_ state precludes direct diffusion-controlled bimolecular radical scavenging, this sharply reduced IP highlights its remarkable transient potential as a “super-electron-donor”. Regarding the specific scavenging pathways, thermodynamic analysis confirmed that HAT and RAF represent the thermodynamically favored pathways, whereas the SET mechanism is thermodynamically unfavorable in comparison. Finally, molecular docking further substantiated that both compounds target the Nrf2-binding site of Keap1, with trans-PTE exhibiting the most robust and potent binding affinity. Collectively, these results suggest that the superior antioxidant efficacy of PTE may arise from a potential synergy between its isomers: the trans-form appears more predisposed to act as a potential Keap1 inhibitor for indirect regulation; meanwhile, the photo-induced cis-form (particularly in the S_1_ state) exhibits enhanced electronic reactivity, implying it may play a crucial role in direct free radical scavenging.

## Figures and Tables

**Table 1:** Thermodynamic parameters of PTE and RES for three radical scavenging mechanisms: HAT, RAF, and SET.

	HAT		SET	RAF		
		ΔG_HAT_	ΔG_SET_			ΔG_RAF_
cis-RES	−H_1_O_1_ + HOO^•^	−1.32	47.27	cis-RES	C_1_ + HOO^•^	−13.01
	−H_2_O_2_ + HOO^•^	2.67			C_2_ + HOO^•^	−11.87
	−H_3_O_3_ + HOO^•^	3.33		trans-RES	C_1_ + HOO^•^	−7.15
trans-RES	−H_1_O_1_ + HOO^•^	−2.24	45.61		C_2_ + HOO^•^	−8.98
	−H_2_O_2_ + HOO^•^	3.28		cis-PTE	C_1_ + HOO^•^	−11.08
	−H_3_O_3_ + HOO^•^	3.10			C_2_ + HOO^•^	−12.61
cis-PTE	−H_1_O_1_ + HOO^•^	−1.56	46.29	trans-PTE	C_1_ + HOO^•^	−9.33
trans-PTE	−H_1_O_1_ + HOO^•^	−2.42	44.72		C_2_ + HOO^•^	−8.62

## Data Availability

The crystal structure data of the Keap1-Nrf2 complex presented in this study are available in the public domain Protein Data Bank (PDB) under accession code 2FLU. This resource is freely available at https://www.rcsb.org/structure/2FLU (accessed on 1 November 2025). All other data generated or analyzed during this study are included in this article (and its Supplementary Information files).

## Supplementary Materials

The following supporting information can be downloaded at: https://www.mdpi.com/article/10.3390/antiox15030325/s1. Table S1. The transition properties of cis-trans isomers of PTE and RES, including maximum absorption wavelengths and oscillator strengths. Table S2. The Fukui function of target molecules in the S_0_ state. Table S3. Global descriptive parameters for all molecules in their S_0_ and S_1_ states. Figure S1. Active space orbital composition for the optimized S_1_/S_0_ CI geometry of RES. Figure S2. Active space orbital composition for the optimized S_1_/S_0_ CI geometry of PTE. Figure S3. Molecular docking simulation illustrating the predicted binding mode of cis-PTE within the active site of the Keap1 protein. Figure S4. Molecular docking simulation illustrating the predicted binding mode of trans-RES within the active site of the Keap1 protein. Figure S5. Molecular docking simulation illustrating the predicted binding mode of cis-RES within the active site of the Keap1 protein.
