# Supplementary material for: Reshaping Antioxidant Activity via Photoisomerization: A Comparative Theoretical Study of Pterostilbene and Resveratrol
Source: Antioxidants (Basel). 2026 Mar 5;15(3):325. doi: 10.3390/antiox15030325 (PMC13024228; doi:10.3390/antiox15030325)
Supplement: Supplementary file 1 [file antioxidants-15-00325-s001.zip › antioxidants-4160380-supplementary.pdf]

Reshaping Antioxidant Activity via Photoisomerization: A Comparative Theoretical Study of Pterostilbene and Resveratrol

Lei Wang and Chaofan Sun *

College of Science, Northeast Forestry University, Harbin 150040, China

*Corresponding author: Chaofan Sun
E-mail address: cfsun@nefu.edu.cn

Captions:

Table S1. The transition properties of cis-trans isomers of PTE and RES, including maximum absorption wavelengths and oscillator strengths.

Table S2. The Fukui function of target molecules in the S_0 state.

Table S3. Global descriptive parameters for all molecules in their S_0 and S_1 states.

Figure S1. Active space orbital composition for the optimized S_1/S_0 CI geometry of RES.

Figure S2. Active space orbital composition for the optimized S_1/S_0 CI geometry of PTE

Figure S3. Molecular docking simulation illustrating the predicted binding mode of cis-PTE within the active site of the Keap1 protein.

Figure S4. Molecular docking simulation illustrating the predicted binding mode of trans-RES within the active site of the Keap1 protein.

Figure S5. Molecular docking simulation illustrating the predicted binding mode of cis-RES within the active site of the Keap1 protein.

Table S1. The transition properties of cis-trans isomers of PTE and RES, including maximum absorption wavelengths and oscillator strengths.

	State	λ_{abs}(nm)	E(eV)	Contribution MO	Strength(f)
cis-PTE	S_1	304.20 nm	4.0758	(68.725%)H→L	0.4474
	S_2	272.81 nm	4.5447	(61.534%)H-1→L	0.0956
	S_3	256.36 nm	4.8363	(54.293%)H→L+1	0.1136
	S_4	231.88 nm	5.3470	(64.887%)H-2→L	0.1367
	S_5	215.13 nm	5.7631	(31.728%)H-3→L	0.4271
	S_6	208.58 nm	5.9442	(56.495%)H→L+2	0.0773
trans-PTE	S_1	317.29 nm	3.9076	(68.725%)H→L	1.1547
	S_2	275.25 nm	4.5045	(62.259%)H-1→L	0.0482
	S_3	260.27 nm	4.7637	(56.865%)H→L+1	0.0567
	S_4	230.86 nm	5.3705	(62.016%)H-2→L	0.0129
	S_5	210.83 nm	5.8807	(50.749%)H→L+2	0.3433
	S_6	209.08 nm	5.9301	(55.288%)H-3→L	0.1748
cis-Res	S_1	299.74 nm	4.1364	(68.787%)H→L	0.4819
	S_2	270.72 nm	4.5799	(61.180%)H-1→L	0.0851
	S_3	255.94 nm	4.8442	(54.913%)H→L+1	0.1034
	S_4	227.25 nm	5.4559	(63.670%)H-2→L	0.1250
	S_5	213.36 nm	5.8110	(32.194%)H-3→L	0.3642
	S_6	207.24 nm	5.9827	(53.687%)H→L+2	0.0760
trans-Res	S_1	313.04 nm	3.9606	(69.187%)H→L	1.1596
	S_2	275.63 nm	4.4981	(62.597%)H-1→L	0.0417
	S_3	259.71 nm	4.7739	(57.674%)H→L+1	0.0610
	S_4	225.13 nm	5.5072	(59.710%)H-1→L	0.0053
	S_5	208.94 nm	5.9341	(43.695%)H→L+2	0.4167
	S_6	208.09 nm	5.9581	(46.172%)H-3→L	0.0208

Table S2. The Fukui function of target molecules in the S_0 state.

		f-	f+	f^0
cis-PTE	$C_{5'}$	0.0216	0.0274	0.0245
	$C_{6'}$	0.0716	0.0627	0.0671
	$C_{7'}$	0.0282	0.0312	0.0297
	$C_{8'}$	0.0295	0.0449	0.0372
	$C_{3'}$	0.0314	0.0447	0.038
	$C_{4'}$	0.0377	0.0391	0.0384
	C_2	0.077	0.0895	0.0833
	O_3	0.016	0.0149	0.0155
	O_2	0.0247	0.0157	0.0202
	C_1	0.0674	0.0976	0.0825
	C_3	0.0488	0.0291	0.0389
	C_4	0.0411	0.039	0.0401
	C_5	0.0444	0.0295	0.037

	C_6	0.0554	0.0484	0.0519
	C_7	0.0359	0.026	0.031
	C_8	0.0374	0.039	0.0382
	O_1	0.0567	0.0306	0.0436
trans-PTE	$C_{7'}$	0.0233	0.0262	0.0247
	$C_{6'}$	0.0755	0.0625	0.069
	$C_{5'}$	0.0245	0.0299	0.0272
	$C_{4'}$	0.0385	0.0443	0.0414
	$C_{3'}$	0.0322	0.0439	0.0381
	$C_{8'}$	0.0379	0.0447	0.0413
	C_2	0.0788	0.0881	0.0835
	C_1	0.0693	0.0958	0.0826
	C_3	0.0466	0.0326	0.0396
	C_4	0.0435	0.0402	0.0419
	C_5	0.0393	0.0294	0.0344
	C_6	0.0556	0.0517	0.0537
	C_7	0.0371	0.0274	0.0323
	C_8	0.043	0.0502	0.0466
	O_1	0.0549	0.0326	0.0438
	O_2	0.0174	0.0137	0.0156
	O_3	0.0189	0.015	0.0169
cis-res	$C_{7'}$	0.0311	0.0309	0.031
	$C_{6'}$	0.057	0.0588	0.0579
	$C_{5'}$	0.0206	0.0294	0.025
	$C_{4'}$	0.0467	0.039	0.0429
	$C_{3'}$	0.0254	0.0436	0.0345
	$C_{8'}$	0.0232	0.0451	0.0342
	C_3	0.0583	0.0307	0.0445
	C_8	0.0395	0.0367	0.0381
	C_7	0.0403	0.0276	0.034
	C_6	0.0607	0.0492	0.0549
	C_5	0.0515	0.029	0.0402
	C_4	0.0451	0.0421	0.0436
	C_2	0.0757	0.0948	0.0852
	C_1	0.0591	0.1005	0.0798
	O_2	0.0315	0.0212	0.0264
	O_3	0.0174	0.0209	0.0192
	O_1	0.0655	0.0307	0.0481
trans-res	$C_{4'}$	0.044	0.0479	0.046
	$C_{5'}$	0.023	0.0312	0.0271
	$C_{6'}$	0.0655	0.059	0.0623
	$C_{7'}$	0.0228	0.027	0.0249
	$C_{8'}$	0.0359	0.045	0.0405
	$C_{3'}$	0.0289	0.0438	0.0363

	C_2	0.0853	0.0902	0.0877
	C_1	0.0693	0.097	0.0831
	C_3	0.0516	0.0334	0.0425
	C_4	0.046	0.0409	0.0434
	C_5	0.0428	0.03	0.0364
	C_6	0.0596	0.0526	0.0561
	C_7	0.0401	0.0279	0.034
	C_8	0.0456	0.0511	0.0483
	O_1	0.0597	0.0332	0.0464
	O_3	0.019	0.0207	0.0199
	O_2	0.0194	0.019	0.0192

Table S3. Global descriptive parameters for all molecules in their S_0 and S_1 states.

		IP	EA	μ	η	S	ω
cis-PTE	S_0	5.37	1.31	-3.34	4.06	0.25	1.38
	S_1	4.66	2.04	-3.35	2.62	0.38	2.14
trans-PTE	S_0	5.23	1.49	-3.36	3.74	0.27	1.51
	S_1	4.95	1.81	-3.38	3.15	0.32	1.82
cis-res	S_0	5.50	1.01	-3.26	4.49	0.22	1.18
	S_1	5.42	1.29	-3.36	4.13	0.24	1.37
trans-res	S_0	5.29	1.48	-3.38	3.81	0.26	1.50
	S_1	5.00	1.81	-3.41	3.19	0.31	1.82

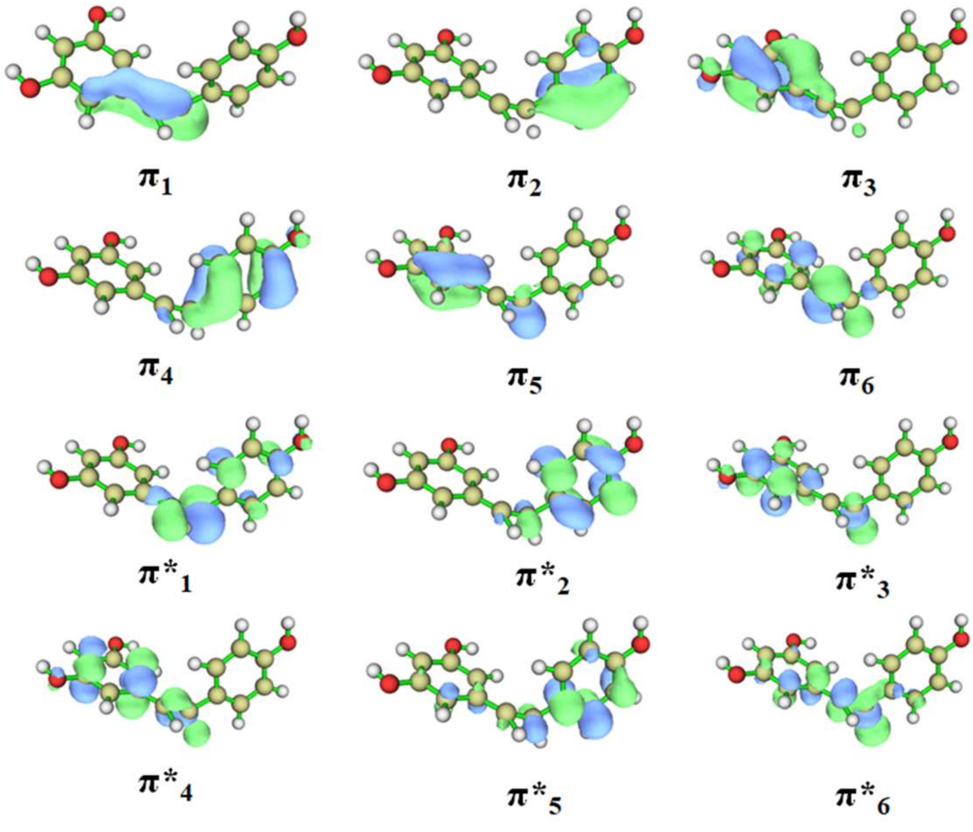

Figure S1. Active space orbital composition for the optimized S_1/S_0 CI geometry of

RES.

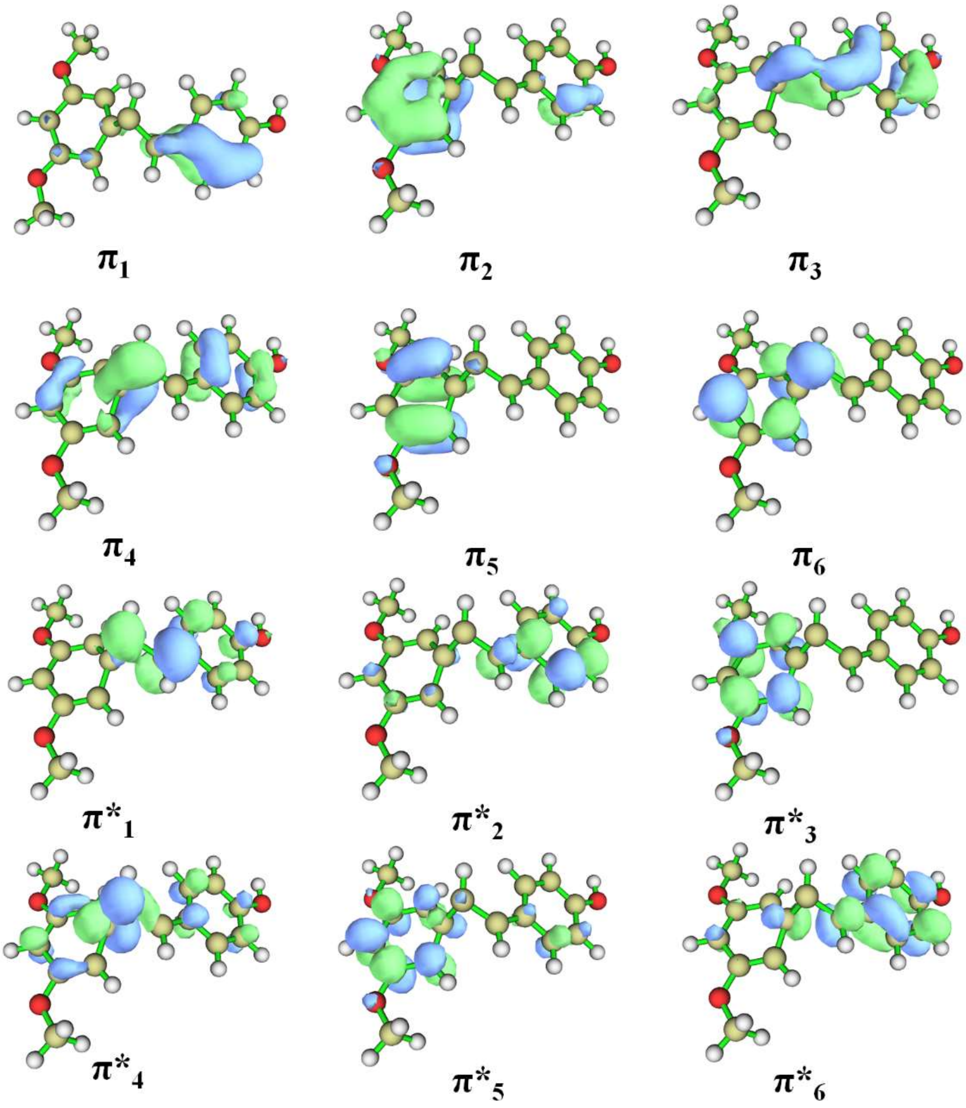

Figure S2. Active space orbital composition for the optimized S_1/S_0 CI geometry of PTE.

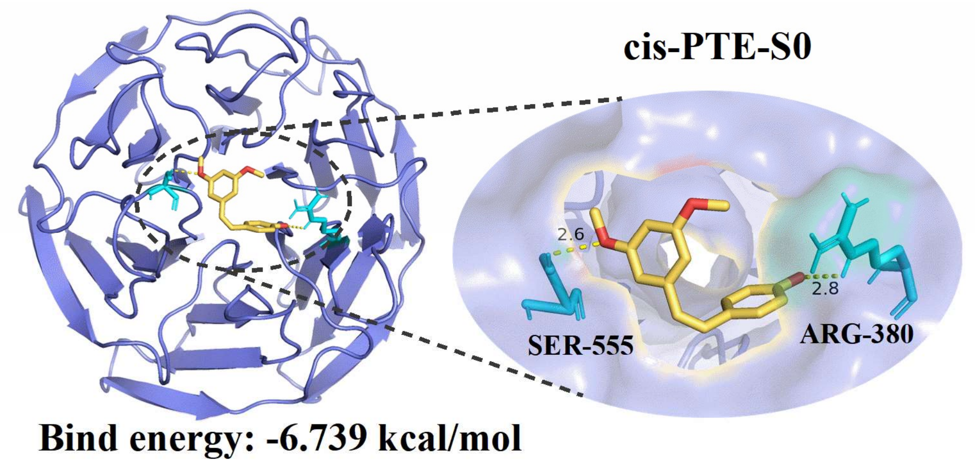

Figure S3. Molecular docking simulation illustrating the predicted binding mode of cis-PTE within the active site of the Keap1 protein.

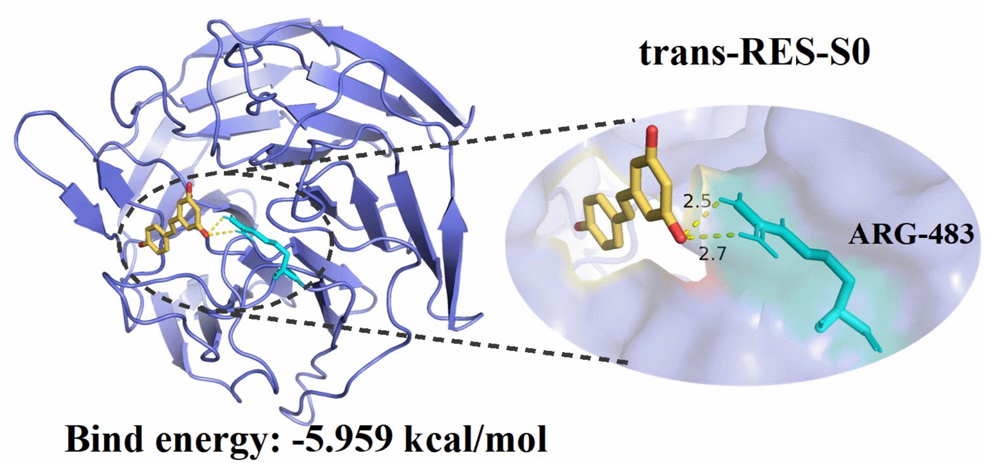

Figure S4. Molecular docking simulation illustrating the predicted binding mode of trans-RES within the active site of the Keap1 protein.

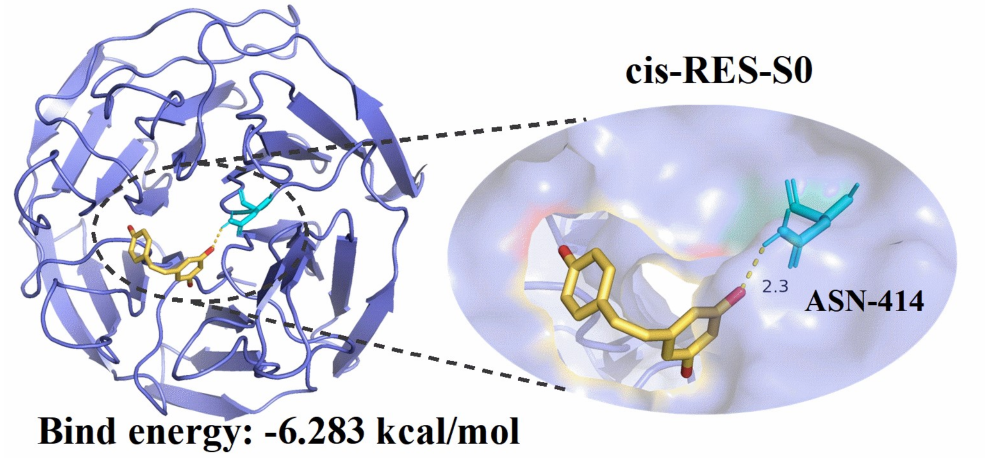

Figure S5. Molecular docking simulation illustrating the predicted binding mode of cis-RES within the active site of the Keap1 protein.
